# Multiple Receptors Contribute to the Attractive Response of Caenorhabditis elegans to Pathogenic Bacteria

**DOI:** 10.1128/spectrum.02319-22

**Published:** 2022-12-13

**Authors:** Wanli Cheng, Hua Xue, Xue Yang, Dian Huang, Minmin Cai, Feng Huang, Longyu Zheng, Donghai Peng, Linda S. Thomashow, David M. Weller, Ziniu Yu, Jibin Zhang

**Affiliations:** a State Key Laboratory of Agricultural Microbiology, National Engineering Research Center of Microbial Pesticides, College of Life Science and Technology, Huazhong Agricultural University, Wuhan, Hubei, China; b Hubei Hongshan Laboratory, Huazhong Agricultural University, Wuhan, Hubei, China; c U.S. Department of Agriculture, Agricultural Research Service, Wheat Health, Genetics and Quality Research Unit, Pullman, Washington, USA; University of Wisconsin—Madison

**Keywords:** *Paenibacillus polymyxa*, *Caenorhabditis elegans*, furfural acetone, attractive olfactory, SRA-13

## Abstract

Nematodes feed mainly on bacteria and sense volatile signals through their chemosensory system to distinguish food from pathogens. Although nematodes recognizing bacteria by volatile metabolites are ubiquitous, little is known of the associated molecular mechanism. Here, we show that the antinematode bacterium Paenibacillus polymyxa KM2501-1 exhibits an attractive effect on Caenorhabditis elegans via volatile metabolites, of which furfural acetone (FAc) acts as a broad-spectrum nematode attractant. We show that the attractive response toward FAc requires both the G-protein-coupled receptors STR-2 in AWC neurons and SRA-13 in AWA and AWC neurons. In the downstream olfactory signaling cascades, both the transient receptor potential vanilloid channel and the cyclic nucleotide-gated channel are necessary for FAc sensation. These results indicate that multiple receptors and subsequent signaling cascades contribute to the attractive response of C. elegans to FAc, and FAc is the first reported ligand of SRA-13. Our current work discovers that P. polymyxa KM2501-1 exhibits an attractive effect on nematodes by secreting volatile metabolites, especially FAc and 2-heptanone, broadening our understanding of the interactions between bacterial pathogens and nematodes.

**IMPORTANCE** Nematodes feed on nontoxic bacteria as a food resource and avoid toxic bacteria; they distinguish them through their volatile metabolites. However, the mechanism of how nematodes recognize bacteria by volatile metabolites is not fully understood. Here, the antinematode bacterium Paenibacillus polymyxa KM2501-1 is found to exhibit an attractive effect on Caenorhabditis elegans via volatile metabolites, including FAc. We further reveal that the attractive response of C. elegans toward FAc requires multiple G-protein-coupled receptors and downstream olfactory signaling cascades in AWA and AWC neurons. This study highlights the important role of volatile metabolites in the interaction between nematodes and bacteria and confirms that multiple G-protein-coupled receptors on different olfactory neurons of C. elegans can jointly sense bacterial volatile signals.

## INTRODUCTION

The survival of animals and plants depends on their ability to sense signal molecules in the environment and then respond appropriately (1–3). For example, gregarious locusts emit an aggregation pheromone, 4-vinylanisole, to attract other locusts and form large-scale swarms (4). The female Aedes aegypti mosquito specializes in seeking and biting the human host by sensing enriched decanal and undecanal from unique human skin lipids (5). *Arabidopsis* metabolizes the ascaroside ascr#18, a pheromone secreted by plant-parasitic nematodes, to generate chemical signals that repel nematodes and reduce infection (6). Nematodes are very common organisms living in the soil, but little is known of how nematodes distinguish among the same species, pathogens, and food resources in the complex soil environment. Some nematodes were reported to recognize each other by the perception of nonvolatile compounds after direct contact; for instance, the predatory nematode Pristionchus pacificus senses the small peptide SELF-1 upon nose contact to avoid self-cannibalism (7), and Caenorhabditis elegans senses signal molecule sulfolipids from predatory nematodes by surface touching and then induces defensive responses (3). Nematodes feed mainly on bacteria, and bacteria in the soil can produce a large number of volatile substances (8, 9). These volatile odors sometimes act as pheromones for animals (10–12); nematodes can also recognize these volatile signals of bacteria to distinguish them as food resources or pathogens. For example, some nematodes distinguish nonpathogenic bacteria from pathogenic bacteria by sensing their volatile metabolite isoamyl alcohol (13); C. elegans exhibits a flight-or-fight response to the pathogenic bacterium Pseudomonas aeruginosa PA14 by sensing its volatile metabolite 1-undecene (14). Although nematodes recognizing bacteria through their volatile metabolites are ubiquitous, little is known of the molecular mechanism of how nematodes distinguish bacteria by sensing their volatile metabolites.

The free-living nematode C. elegans is a good model in which to evaluate chemosensory mechanisms and related behaviors owing to the detailed knowledge of its nervous system. C. elegans feeds mainly on bacteria and distinguishes food from pathogens in its food-searching behavior by its highly developed chemosensory system (15). The highly developed chemosensory system of C. elegans comprises 12 pairs of amphid chemosensory neurons, which enable it to detect a wide variety of signals associated with food, danger, or other animals (16, 17). The detection of attractive volatile organic compounds (VOCs) is mediated mainly by the AWA and AWC olfactory neurons (16–19). Most chemosensory odorant receptors in C. elegans are G-protein-coupled receptors (GPCRs) (20). The genome of C. elegans is predicted to harbor at least 1,300 GPCR genes (21), accounting for approximately 7% of its protein-encoding genes (22), but only a few GPCRs of olfactory neurons have known specific ligands (18–20, 23). The individual sensory receptors that respond to ligands and the subsequent signaling cascades that they initiate in the olfactory neurons of C. elegans still require further elucidation.

Paenibacillus polymyxa KM2501-1 was isolated from the rhizosphere soil of buttercups (*Ranunculus*) and was reported to exhibit nematicidal activity *in vitro* and control efficiency against Meloidogyne incognita in nematode-infected tomatoes in pot experiments (24, 25). In this study, the antinematode bacterium P. polymyxa KM2501-1 was found to exhibit an attractive effect on nematodes via its volatile metabolites. *P. polymyxa* KM2501-1 and C. elegans were then used to study the molecular mechanism of nematodes perceiving bacterial volatile signals and inducing an attraction response.

## RESULTS

### The pathogenic bacterium *P. polymyxa* KM2501-1 elicits an attractive response in C. elegans via volatile metabolites.

**FIG 1:**
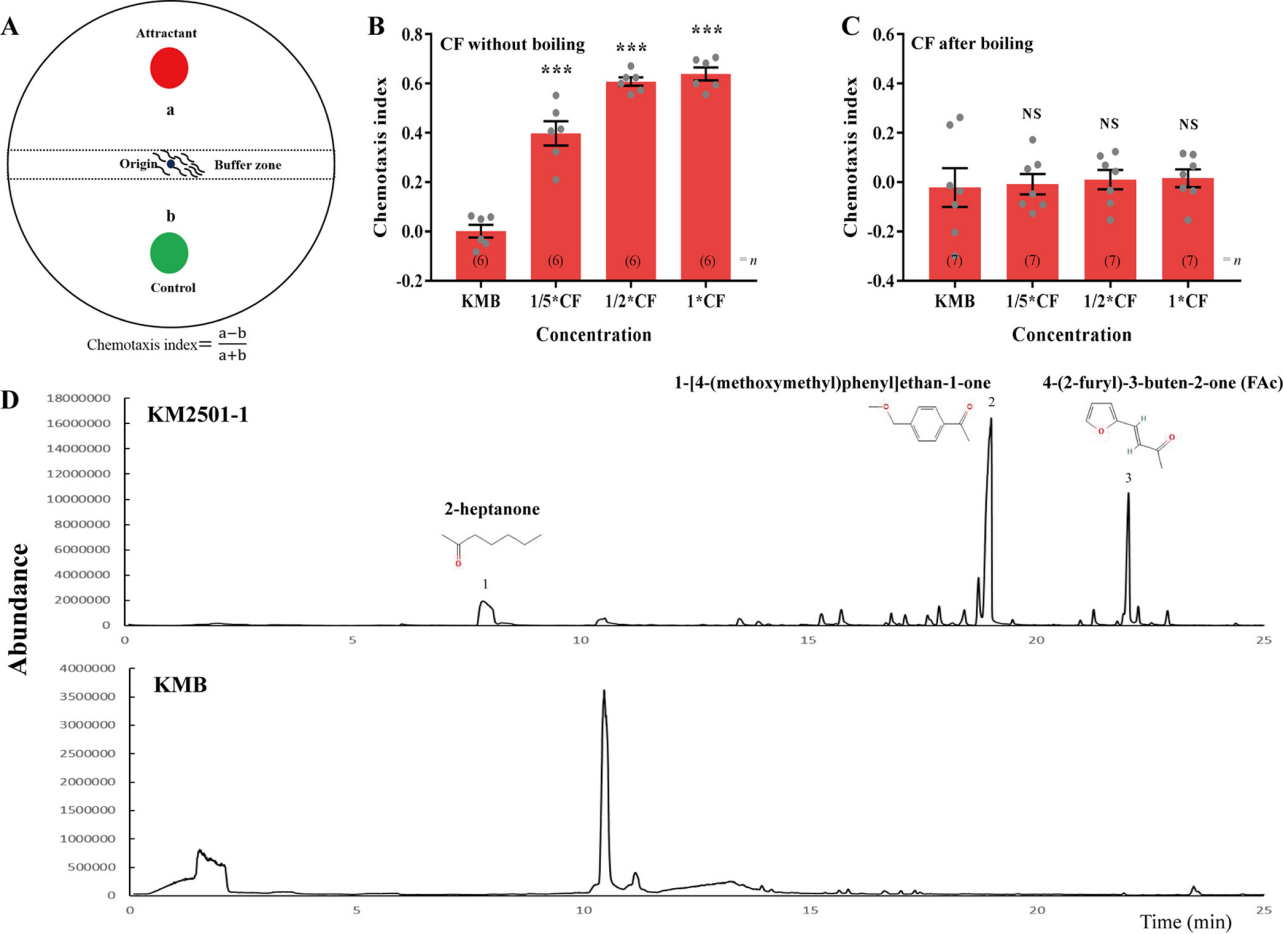
The pathogenic bacterium *P. polymyxa* KM2501-1 elicits an attractive response in C. elegans via volatile metabolites. (A) Schematic diagram of the chemotaxis assay. A thin layer of 2% water agar in a 9-cm petri dish was used as the substrate for chemotaxis since nematodes move efficiently over the agar surface. About 200 washed nematodes were placed at the center of the dish with an 11.2-mm-diameter filter paper wetted with 30 μL of a chemical solution or the culture filtrate (CF) of *P. polymyxa* KM2501-1 on one side of the plate (attractant area) and another 11.2-mm-diameter filter paper wetted with 30 μL of ethanol or KMB broth on the opposite side (control area). The distance between the center of each filter paper and the midline of the plate was 25.6 mm. Chemotaxis assays were performed at 20°C for 2 h in the dark, and the numbers of nematodes in the attractant and control areas were counted to calculate the chemotaxis index. (B) Chemotactic response of wild-type C. elegans toward the CF of *P. polymyxa* KM2501-1 at various dilutions. (C) Chemotactic response of wild-type C. elegans toward various dilutions of the *P. polymyxa* KM2501-1 CF after boiling in water for 30 min. (D) GC-MS chromatograms of KMB broth and fermentation broth of KM2501-1. Error bars indicate means ± SEM. ***, *P < *0.001; NS, not significant (two-tailed unpaired Student’s *t* test was used for statistical comparison between the values of the treatments and the control [KMB]).

*P. polymyxa* KM2501-1 has been reported to have nematicidal activity, but its chemotactic effect on nematodes remains unknown. In order to systematically study the chemotactic response in C. elegans toward *P. polymyxa* KM2501-1, an experimental device (Fig. 1A) was designed to check the behavior of worms toward the culture filtrate (CF) of strain KM2501-1. Nematodes prefer the CF of strain KM2501-1 to Kings medium B (KMB) broth, with chemotaxis indices of 0.398, 0.608, and 0.639 at concentrations of 1/5×, 1/2×, and 1×, respectively, while the chemotaxis index of the control group (KMB) was only 0.001 (Fig. 1B). Interestingly, the CF of strain KM2501-1 no longer exhibited attractive activity on nematodes after boiling to remove volatile substances (Fig. 1C). In order to confirm the activity of volatile metabolites of strain KM2501-1 in attracting nematodes further, activated charcoal was used to remove the volatile substances in the CF of strain KM2501-1 in a three-compartment petri plate device (see Fig. S1A in the supplemental material). Compared with the CF of strain KM2501-1 without treatment with activated charcoal, the attractive activity of the CF on nematodes decreased significantly after the removal of volatiles by activated charcoal (Fig. S1B). Taken together, these results indicate that *P. polymyxa* KM2501-1 elicits an attractive response in C. elegans via volatile metabolites. Next, a solid-phase microextraction–gas chromatography-mass spectrometry (SPME-GC-MS) analysis was conducted to identify the VOCs produced by *P. polymyxa* KM2501-1. Apart from the peaks presented in the chromatograms for KMB broth, in total, 3 main peaks whose area percentages were above 5% were present in the chromatograms of the strain KM2501-1 fermentation broth. These 3 main peaks were then identified as 2-heptanone, 1-[4-(methoxymethyl)phenyl]ethan-1-one, and 4-(2-furyl)-3-buten-2-one, respectively, by GC-MS (Fig. 1D). Among them, 2-heptanone has been reported to stimulate the attraction response of C. elegans by binding with the GPCR STR-2 of AWC neurons (18), 1-[4-(methoxymethyl)phenyl]ethan-1-one was not commercially available, and 4-(2-furyl)-3-buten-2-one, also known as furfural acetone (FAc), was used to test its chemotactic effect on nematodes further.

### The volatile metabolite FAc of *P. polymyxa* KM2501-1 acts as a broad-spectrum attractant of nematodes.

**FIG 2:**
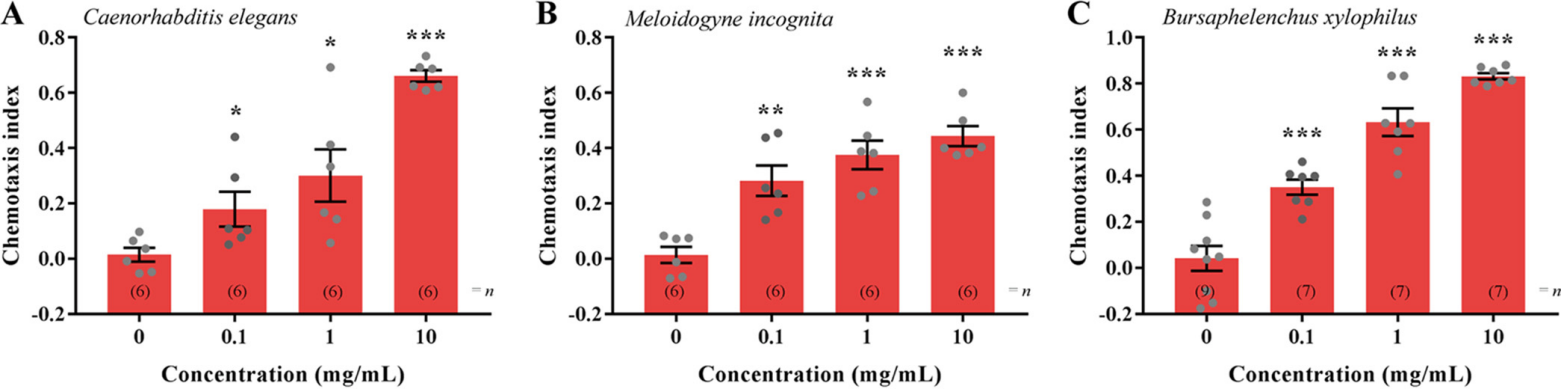
Chemotactic response of wild-type C. elegans (A), Meloidogyne incognita (B), and *Bursaphelenchus xylophilus* (C) to FAc at various concentrations. Error bars indicate means ± SEM. *, *P < *0.05; **, *P < *0.01; ***, *P < *0.001 (two-tailed unpaired Student’s *t* test was used for statistical comparison between the values of the treatments and the control [0 mg/mL]).

According to the results of chemotaxis assays, FAc exhibits a significant attractive effect on C. elegans at concentrations of 0.1 to 10 mg/mL, among which the attractive activity is the strongest at a concentration of 10 mg/mL, with a chemotaxis index of 0.661 (Fig. 2A). These results suggest that *P. polymyxa* KM2501-1 exhibits an attractive effect on C. elegans mainly via its volatile metabolites 2-heptanone and FAc. Interestingly, FAc at a concentration of >0.1 mg/mL also has significant nematicidal activity against C. elegans (Fig. S2), indicating that the volatile metabolite FAc of *P. polymyxa* KM2501-1 exhibits dual attract-and-kill effects on C. elegans at concentrations of 0.1 to 10 mg/mL. In addition, FAc at various concentrations also exhibits significant attractive activity on two species of plant-pathogenic nematodes, Meloidogyne incognita and Bursaphelenchus xylophilus (Fig. 2B and C), suggesting that FAc may act as a broad-spectrum nematode attractant that can be used to control nematodes in the agricultural industry.

### The GPCRs SRA-13 of AWA neurons and SRA-13 and STR-2 of AWC neurons are required for sensing FAc.

**FIG 3:**
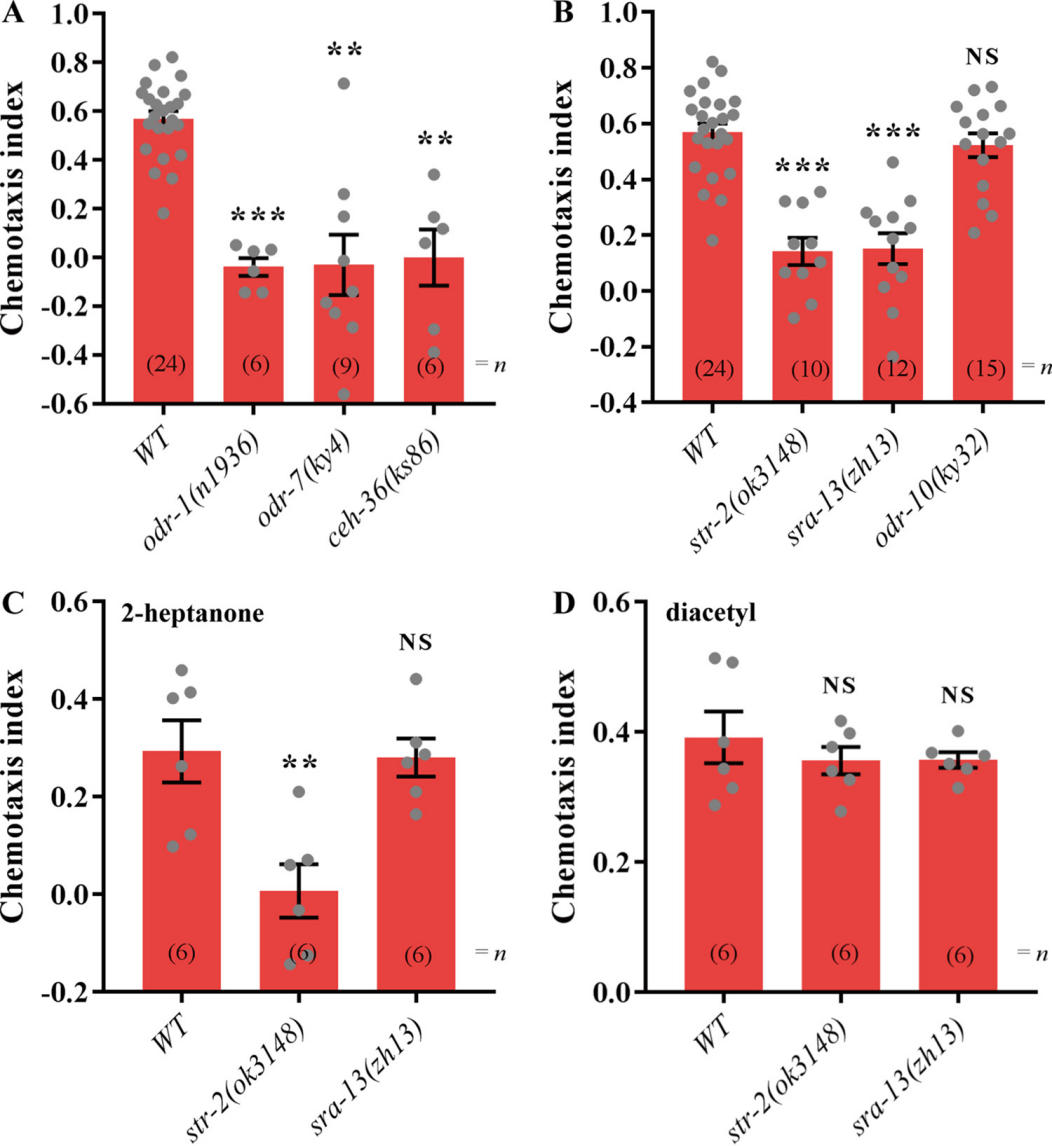
Chemotaxis to FAc depends on AWA and AWC olfactory neurons and the GPCRs SRA-13 and STR-2 expressed on them. (A) Chemotaxis effects of FAc at 10 mg/mL on wild-type (WT), *odr-1* (*n1936*), *ceh-36* (*ks86*), and *odr-7* (*ky4*) animals. (B) Chemotaxis effects of FAc at 10 mg/mL on WT, *str-2* (*ok3148*), *odr-10* (*ky32*), and *sra-13* (*zh13*) animals. (C) Chemotaxis effects of 2-heptanone at 1 mg/mL on WT, *str-2* (*ok3148*), and *sra-13* (*zh13*) animals. (D) Chemotaxis effect of diacetyl at 10 mg/mL on WT, *str-2* (*ok3148*), and *sra-13* (*zh13*) animals. Error bars indicate means ± SEM. **, *P < *0.01; ***, *P < *0.001; NS, not significant (two-tailed unpaired Student’s *t* test was used for statistical comparisons of the WT to the mutants).

Two pairs of olfactory neurons, AWA and AWC, are responsible for detecting attractive odorants in C. elegans (16, 17, 23). Because of the attractive response of nematodes to FAc odorants, we assumed that at least one FAc-responsive receptor is expressed in AWA or AWC olfactory neurons. In order to test this assumption, C. elegans mutants with known defects in cell specification or cell-specific components of the olfactory transduction pathway were used in this study. *odr-7* (*ky4*) mutants failed to respond to the odorants normally detected by AWA neurons, while their AWC function was intact (26, 27), and *ceh-36* mutants failed to respond to any AWC-sensed odorants (27–29). The *odr-1* gene is expressed in AWB, AWC, and other neurons but not AWA neurons; *odr-1* mutants are therefore defective in AWC- and AWB-mediated olfaction (27, 30). Hence, the chemotaxis indices of *odr-7*, *ceh-36*, or *odr-1* mutants for FAc were measured. The results showed that none of the *odr-1*, *ceh-36*, or *odr-7* mutants were attracted to FAc (Fig. 3A), indicating that a defect in either AWA or the AWC olfactory neurons impedes the sensing of FAc.

GPCRs have been reported to be the most common olfactory receptors in C. elegans and mammals (16, 17, 20). Both AWA and AWC olfactory neurons are responsible for detecting FAc odors. We therefore hypothesized that the GPCRs ODR-10 and STR-2, expressed in AWA and AWC neurons, respectively (16–19, 23), and SRA-13, expressed in both AWA and AWC neurons (16), are likely receptors for FAc. Chemotaxis assays revealed that worms with a mutation in *str-2* (*ok3148*) or *sra-13* (*zh13*) were defective in sensing FAc, but nematodes with the *odr-10* (*ky32*) mutation retained a normal response to FAc (Fig. 3B). This result implied that the impaired chemoattraction was due to the loss of function of the GPCRs encoded by *str-2* and *sra-13*.

To verify the specificity between the receptor STR-2 or SRA-13 and its signal molecule FAc, the *str-2* (*ok3148*) and *sra-13* (*zh13*) mutants were tested for chemotaxis to 2-heptanone, the other attractant sensed by the olfactory receptor STR-2 on AWC olfactory neurons, and diacetyl, an attractant sensed by the olfactory receptor ODR-10 on AWA olfactory neurons. Mutation of *str-2* had no effect on worms sensing diacetyl but affected the sensing of 2-heptanone (Fig. 3C and D), which is consistent with a previous report (18). The worms with a mutation of *sra-13* responded normally to 2-heptanone and diacetyl (Fig. 3C and D). These results suggest that the receptor SRA-13 can sense FAc but not diacetyl and 2-heptanone, while the receptor STR-2 can perceive both of the signal molecules FAc and 2-heptanone.

**FIG 4:**
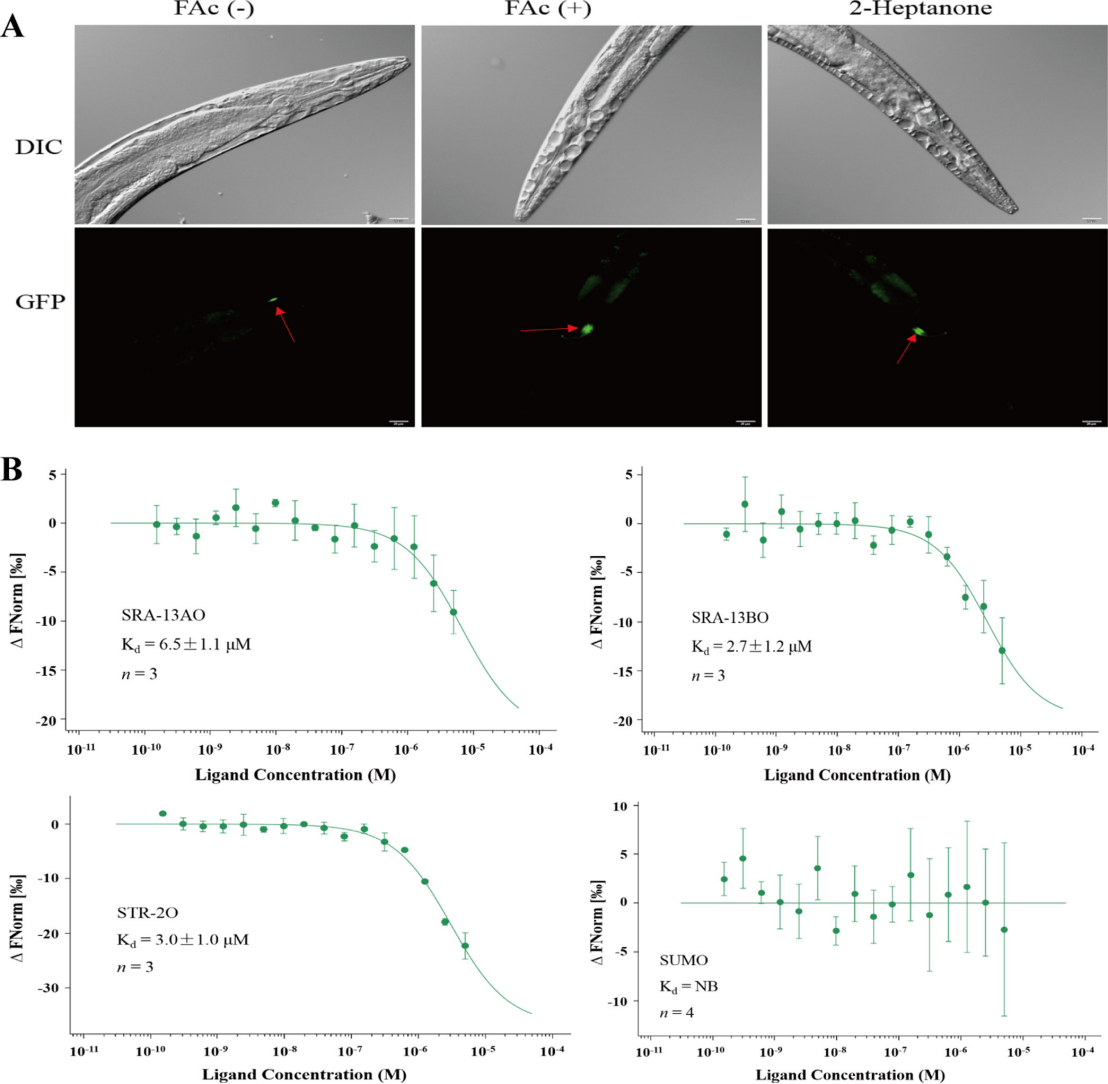
Interaction between FAc and the target GPCR SRA-13 or STR-2. (A) Expression of STR-2 in AWC neurons induced by 2-heptanone or FAc. The nematode mutant strain *kyIs140* [*str-2*::GFP + *lin-15*(+)] was immersed in 2-heptanone, an FAc solution, or distilled water for 45 min, and the changes in the fluorescence were then visualized. DIC, differential interference contrast. The experiment was repeated 3 times, and each treatment included at least 5 nematodes. (B) Dose-response curves of the interaction between FAc and STR-2O, SRA-13AO, SRA-13BO, or a SUMO tag (control) by MST analysis. Error bars indicate means ± standard deviations (SD).

The C. elegans mutant *kyIs140* [*str-2*::GFP (green fluorescent protein) + *lin-15*(+)] was used to determine whether the expression of STR-2 in AWC neurons was induced by FAc. 2-Heptanone has been reported to be a ligand of the GPCR STR-2 (18), and it was used as a positive control in this test. Compared with the control, the fluorescence of the STR-2::GFP mutant in AWC neurons was enhanced after exposure to FAc or 2-heptanone (Fig. 4A).

The GPCRs STR-2 and SRA-13 are transmembrane proteins that are difficult to heterologously express in a soluble form. We therefore predicted the extracellular domains of STR-2 (STR-2O) and SRA-13 isoform A (SRA-13AO) and isoform B (SRA-13BO) using the TMHMM server (Fig. S3). All of the genes of the extracellular domain of the GPCR STR-2 were sequentially linked and synthesized to create the *str-2o* gene, which was then transferred to Escherichia coli for heterologous expression as the STR-2O protein. Similarly, all of the genes of the extracellular domains of GPCR SRA-13 isoform A or B were sequentially linked and synthesized to create the *sra-13ao* or *sra-13bo* gene and heterologously expressed as the SRA-13A or SRA-13BO protein in E. coli, respectively. These proteins were then purified using Ni-nitrilotriacetic acid (NTA) (Fig. S4). The binding affinities of FAc for STR-2O, SRA-13AO, and SRA-13BO were measured by microscale thermophoresis (MST). The dissociation constant (*K_d_*) values for FAc binding to STR-2O, SRA-13AO, and SRA-13BO were 3.0 μM, 6.5 μM, and 2.7 μM, respectively, whereas no binding affinity was detected between FAc and the control SUMO tag (Fig. 4B), indicating the affinity of FAc for the GPCRs STR-2 and SRA-13, which verified the hypothesis that they are target proteins of FAc in AWA and AWC olfactory neurons.

### FAc activates the PLC pathway and the cGMP pathway of C. elegans.

**FIG 5:**
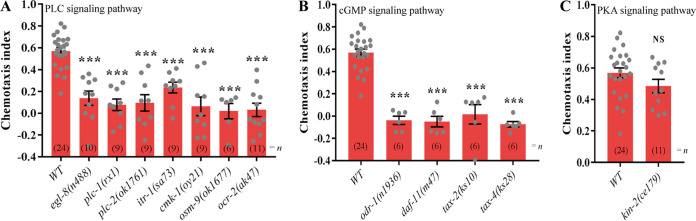
The chemotactic response of wild-type (WT) C. elegans and mutants of genes in the PLC signaling pathway (A), the cGMP signaling pathway (B), and the PKA signaling pathway (C) to 10 mg/mL FAc. Error bars indicate means ± SEM, ***, *P < *0.001; NS, not significant (two-tailed unpaired Student’s *t* test was used for statistical comparisons of the WT to the mutants).

The stimulation of GPCRs in AWA or AWC olfactory neurons can activate certain signaling cascades and ion channels (16–18, 20). In C. elegans, the phospholipase C (PLC) pathway and the transient receptor potential vanilloid (TRPV) channel are required for chemotaxis to odors detected by AWA neurons, whereas the cGMP pathway and the cyclic nucleotide-gated (CNG) channel are involved in sensing odors by AWC neurons (17). We therefore screened mutants in *egl-8*, *plc-1*, *plc-2*, *itr-1*, *cmk-1*, *osm-9*, and *ocr-2*, which are involved in the PLC pathway and the TRPV channel, for their effects on chemotaxis to FAc. The results showed that the attractive responses of mutants in the genes *egl-8* (*n488*), *plc-1* (*rx1*), *plc-2* (*ok1761*), *itr-1* (*sa73*), *cmk-1* (*oy21*), *osm-9* (*ok1677*), and *ocr-2* (*ak47*) toward FAc were all reduced significantly compared to the response of the wild-type (WT) nematodes (Fig. 5A). In the cGMP pathway and the CNG channel, mutants in *odr-1* (*n1936*), *daf-11* (*m47*), *tax-2* (*ks10*), and *tax-4* (*ks28*) also affected the sensing of FAc (Fig. 5B). Other pathways such as cAMP-dependent protein kinase (PKA) were also queried, but the *kin-2* (*ce179*) mutant had no effect on the sensing of FAc (Fig. 5C). These results indicate that both the PLC pathway and the cGMP pathway are necessary for signal transduction for nematode sensing of FAc.

## DISCUSSION

In the natural environment, nematodes feed mainly on microorganisms, especially bacterial communities, and bacteria have evolved various defense mechanisms to help them to resist predation by nematodes (31). For example, some *Bacillus* species can produce toxic metabolites to kill nematodes (32, 33), and some Yersinia pestis strains can prevent nematode feeding by controlling biofilm formation (34). *P. polymyxa* KM2501-1 was found to have good nematicidal activity by producing multiple toxic volatile metabolites (24, 25). In this study, *P. polymyxa* KM2501-1 was found to elicit an attractive response in nematodes mainly via the volatile metabolites 2-heptanone and FAc (Fig. 1 and 2). FAc exhibits attractive activity on C. elegans that is equivalent to or even stronger than that of 2-heptanone at the same concentrations (see Fig. S5 in the supplemental material). Interestingly, FAc can act as a broad-spectrum attractant on C. elegans and the plant-pathogenic nematodes M. incognita and B. xylophilus (Fig. 2) and exhibits dual attract-and-kill effects on C. elegans (Fig. 2; Fig. S2) and *M. incognita* (35). These characteristics of FAc that enable its specific molecular mechanism of attracting nematodes are worthy of further study. Here, we use the genetically well-described model nematode C. elegans to investigate the signaling pathways and molecular targets involved in the mechanism of FAc attracting nematodes.

In C. elegans, the binding of odorants to receptors on the external surface of cilia is generally the first step in the initiation of olfactory attraction, with further signal transduction being mediated by AWA or AWC olfactory neurons (16–18, 20, 23). Both AWA and AWC olfactory neurons are responsible for the detection of FAc (Fig. 3A). Hence, the GPCRs STR-2, SRA-13, and ODR-10 expressed in AWA or AWC neurons are candidate receptors for FAc. The GPCR STR-2 is reportedly a receptor of the volatile 2-heptanone in AWC neurons (18). The GPCR SRA-13 is another candidate receptor in AWA and AWC neurons, which affects the olfactory plasticity of C. elegans (16, 36), but its specific ligand is still unknown. We found that mutations in the *str-2* or *sra-13* gene affected the attractive response of C. elegans toward FAc (Fig. 3B), the expression of STR-2 was enhanced when nematodes were exposed to FAc (Fig. 4A), and FAc was bound to the extracellular domains of STR-2 and SRA-13 *in vitro* (Fig. 4B). Collectively, these data indicate that the attractive response of C. elegans toward FAc requires both the GPCR STR-2 in AWC neurons and the GPCR SRA-13 in AWA and AWC neurons. These findings also demonstrate that FAc is the first reported ligand of SRA-13. In the downstream olfactory signal cascades, both the CNG channel and the TRPV channel are necessary for FAc sensing (Fig. 5). Until now, all volatile odors were known to bind to only a single GPCR of C. elegans; for example, 2-heptanone targets the GPCR STR-2 in AWC neurons (18), sex pheromones emitted by C. elegans males specifically bind to the GPCR SRD-1 in AWA neurons of females (20), and diacetyl specifically binds to the GPCR ODR-10 in AWA neurons of C. elegans (23). It is noteworthy that in the present study, we find that a bacterial volatile metabolite, FAc, binds to at least two different GPCRs located in different olfactory neurons and activates multiple downstream olfactory signals to stimulate the attractive response of C. elegans. In addition, Both SRA-13AO and SRA-13BO can bind to FAc (Fig. 4B). Although the sequence similarity of SRA-13AO and SRA-13BO is 48.7% (57/117), there are two segments of these two proteins that match completely (Fig. S6). This indicates that these two fragments may be the key regions for the binding of SRA-13 to FAc.

**FIG 6:**
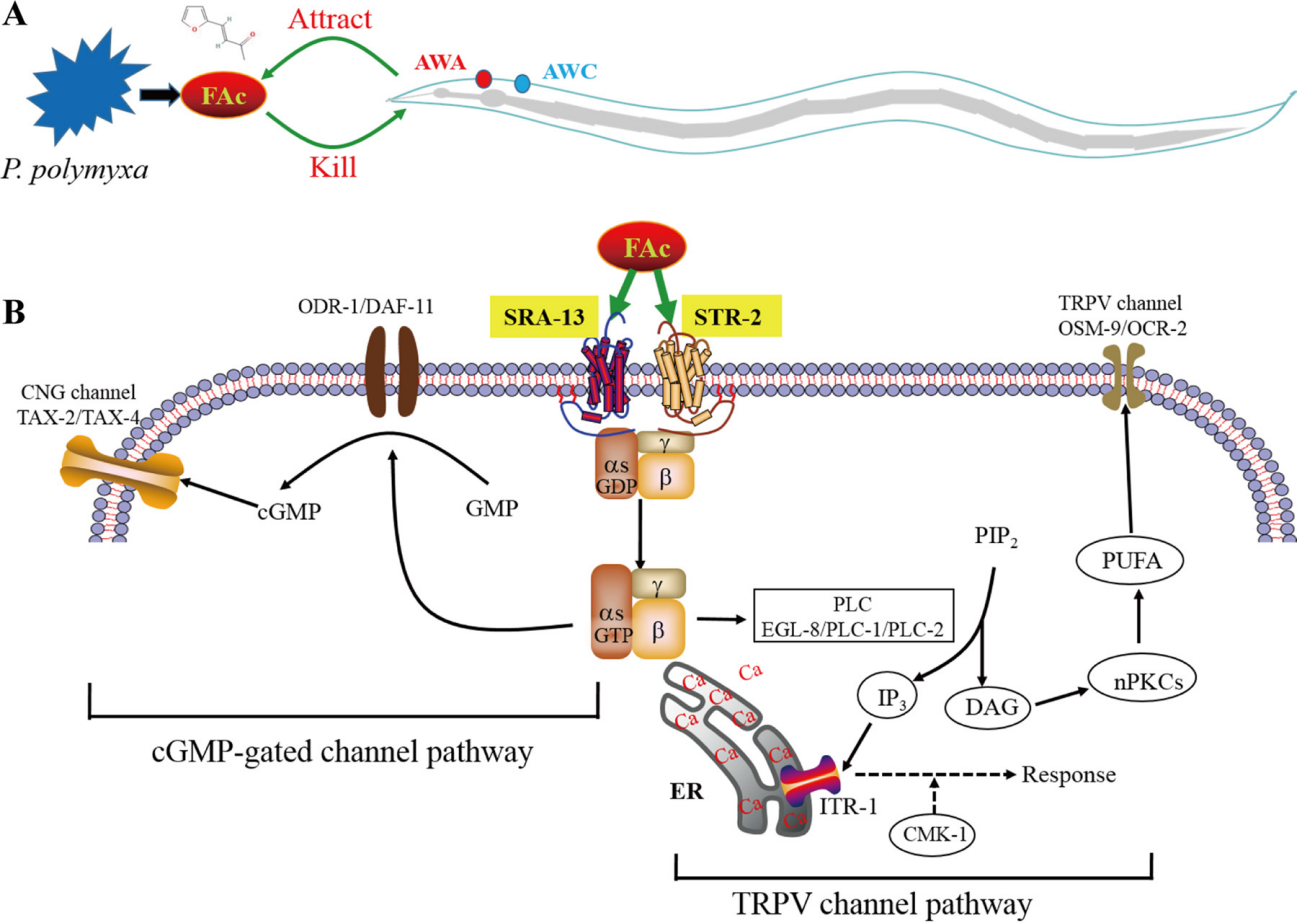
Molecular model of the attractive effect of *P. polymyxa* KM2501-1 on C. elegans. (A) Model of the attract-and-kill effect of *P. polymyxa* KM2501-1 on C. elegans. *P. polymyxa* KM2501-1 emits attractive odors, including FAc, into the environment, which are then detected by the AWA and AWC olfactory neurons of C. elegans. The worms move toward strain KM2501-1 and ingest toxic metabolites, including FAc, leading to the death of the nematodes. (B) Molecular mechanism of the attraction response of C. elegans induced by FAc. FAc targets the GPCR STR-2 in AWC olfactory neurons and the GPCR SRA-13 in AWA and AWC olfactory neurons and then activates the TRPV channel via the PLC pathway and the CNG channel via the cGMP pathway. These iron channels lead to the attractive response of C. elegans.

In summary, we have set a model of the attract-and-kill effect of *P. polymyxa* KM2501-1 on C. elegans via volatile metabolites (Fig. 6A). Volatile metabolites of *P. polymyxa* KM2501-1, including FAc, eliciting attractive responses of C. elegans depend on AWA and AWC neurons. Nematodes move toward strain KM2501-1 and ingest FAc or other toxic metabolites, leading to the death of the nematodes. Here, we also reveal the molecular mechanism of the attraction response of C. elegans induced by FAc (Fig. 6B). FAc odors were emitted into the environment by *P. polymyxa* KM2501-1 and sensed by AWA and AWC olfactory neurons of C. elegans. FAc molecules are detected by the GPCR SRA-13 in AWA neurons and target the GPCRs SRA-13 and STR-2 in AWC neurons. These GPCRs activate downstream Gα subunits (16–18). G proteins then regulate the production of diacylglycerol (DAG) and inositol 1,4,5-trisphosphate (IP3) via the PLC pathway, finally leading to the opening of the TRPV channel (OSM-9/OCR-2) and Ca^2+^ influx (17). G proteins also regulate cGMP production through guanylate cyclase (ODR-1/DAF-11) to open the CNG channel (TAX-2/TAX-4). The opening of the TRPV and CNG channels leads to the attractive response of C. elegans.

Nematodes feed on nontoxic bacteria as a food resource and avoid toxic bacteria; they distinguish them by their chemosensory system (15). In this study, a pathogenic bacterium, *P. polymyxa* KM2501-1, can also attract nematodes. The volatile metabolite FAc emitted by the antinematode bacterium *P. polymyxa* KM2501-1 exhibits dual functions, attracting and killing nematodes (Fig. 2; Fig. S2). The pathogenic bacterium attracts and kills nematodes by secreting volatile metabolites, broadening our understanding of the interaction between microorganisms and nematodes. Although perception systems of nematodes deceived by antinematode microorganisms to lure and subsequently kill them are ubiquitous (37–41), the mechanism by which nematodes are deceived by these toxic microorganisms is poorly understood. In many ecosystems, apart from the signaler and the intended receiver participants involved in deciphering chemical signaling, unintended eavesdroppers may also decipher the chemical signal (42). *P. polymyxa* KM2501-1 is a kind of soil bacterium; its attractant chemical signals FAc and 2-heptanone can also be produced by some plants or other microorganisms in the soil environment (43–45). *P. polymyxa* KM2501-1 may unintentionally eavesdrop on the FAc signal between nematodes and plants or other bacteria, resulting in the dual attract-and-kill functions of strain KM2501-1 on nematodes.

## MATERIALS AND METHODS

### Chemicals.

Furfural acetone (FAc) (purity of >98%), 2-heptanone (purity of >98%), and diacetyl (purity of >98%) were purchased from TCI (Tokyo Chemical Industry) (Japan). These chemicals were dissolved in anhydrous ethanol for use in chemotaxis experiments in this study.

### Nematode culture and strains.

All C. elegans strains were maintained at 20°C on nematode growth medium and fed with Escherichia coli OP50. The N2 Bristol strain of C. elegans was used as the wild type. The mutated and transgenic strains used in this study include *odr-1* (*n1936*), *odr-7* (*ky4*), *ceh-36* (*ks86*), *daf-11* (*m47*), *odr-10* (*ky32*), *str-2* (*ok3148*), *sra-13* (*zh13*), *osm-9* (*ok1677*), *tax-2* (*ks10*), *tax-4* (*ks28*), *ocr-2* (*ak47*), *cmk-1* (*oy21*), *plc-1* (*rx1*), *plc-2* (*0k1761*), *kin-2* (*ce179*), *egl-8* (*n488*), *itr-1* (*sa73*), and *kyIs140* [*str-2*::GFP *lin-15*(*+*)] strains, which were provided by the *Caenorhabditis* Genetics Center. *B. xylophilus* was maintained at 28°C on potato dextrose agar (PDA) medium and fed with Botrytis cinerea. C. elegans and *B. xylophilus* were synchronized as previously described (46), and the synchronized nematodes were used in subsequent experiments. *M. incognita* was maintained on the roots of tomato (Solanum lycopersicum) and grown in a greenhouse at 25°C ± 1°C. The egg masses of *M. incognita* were peeled from the infected tomato roots with needles, washed three times in distilled water, and placed into 24-well culture plates with distilled water at 20°C for 3 days. Freshly hatched J2 juveniles were collected and used immediately for all of the assays.

### Preparation of *P. polymyxa* KM2501-1.

*P. polymyxa* KM2501-1 was isolated from the rhizosphere soil of buttercups (*Ranunculus*) in Hukou County, Jiangxi Province, China (24). Strain KM2501-1 was grown on a KMB agar plate at 28°C for 48 h, and a single colony was inoculated into 100 mL KMB broth and incubated on a rotary shaker (180 rpm) at 28°C in the dark for 24 h as the seed culture. Subsequently, 1 mL of the seed culture was inoculated into 100 mL of KMB broth and incubated on a rotary shaker (180 rpm) at 28°C in the dark for 48 h. Cultures were centrifuged, and the supernatant solution was passed through a 0.22-μm nitrocellulose filter to prepare sterile culture filtrates for the assays described below.

### Chemotaxis assay.

Chemotaxis assays were performed as described previously (24), using 9-cm petri dishes (Fig. 1A). A dish containing 10 mL of 2% water agar was divided into a buffer zone including the 0.8-cm width of the middle line, an attractant area, and a control area. Sterile filter paper discs (11.2-mm diameter) were placed symmetrically in the attractant and control areas, with a distance of 25.6 mm between their center and the midline of the plate. Next, 30 μL of a chemical solution (dissolved in anhydrous ethanol) or the CF of *P. polymyxa* KM2501-1 at different dilutions was spotted onto the filter paper in the attractant area, and the same volume of anhydrous ethanol or KMB broth was spotted onto the control filter paper. Nematodes were washed three times with M9 buffer and once with water to remove bacteria, and approximately 200 synchronized L4 C. elegans larvae, L2 *B. xylophilus* larvae, or J2 juveniles of *M. incognita* were placed in the center of the plate. Chemotaxis assays were performed at 20°C for 2 h in the dark. The numbers of nematodes in the attractant and control areas were then counted under an inverted microscope. Nematodes that remained within the 8-mm buffer zone at the midline of the plate were not counted. The chemotaxis index was calculated as (number of nematodes in the attractant area − number of nematodes in the control area)/(number of nematodes in the attractant area + number of nematodes in the control area). The chemotaxis assays for each compound at each concentration were repeated at least 6 times.

### Nematicidal assay.

The nematicidal assays were performed in 96-well plates. A total of 200 μL of an FAc solution (dissolved in distilled water) and 30 to 40 synchronized L2 C. elegans larvae were transferred to the wells, and distilled water was used as a control. Each treatment had three repetitions. Plates were covered with plastic lids and maintained in the dark at 20°C for 24 h. Worms that did not move when they were gently prodded and that displayed no pharyngeal pumping were considered dead. The experiment was repeated 6 times for each concentration.

### Solid-phase microextraction and gas chromatography-mass spectrometry.

The extraction of VOCs from the fermentation broth of strain KM2501-1 by solid-phase microextraction (SPME) and the identification of these VOCs by gas chromatography-mass spectrometry (GC-MS) were described previously (24). A new 75-mm CAR/PDMS SPME fiber (Supelco, Bellefonte, PA, USA) was conditioned with helium at 270°C for 2 h prior to use. After each extraction cycle, the fiber was returned to the SPME needle to prevent contamination and conditioned again with helium at 270°C for 20 min. Extractions were performed in 15-mL Supelco SPME vials filled with 9 mL of the bacterial culture, and the vials were clamped inside a thermostatic water bath. The SPME needle was allowed to pierce the septum, and the fiber was exposed to the headspace of the vial for 90 min at 60°C with constant magnetic stirring. The VOCs from 9 mL of KMB broth were used as controls. A Hewlett Packard 7890GC/5975MSD instrument (Agilent Technologies, USA) equipped with an HP-5MS capillary column was used to separate and identify the VOCs. The carrier gas was helium at a flow rate of 1 mL/min in split-splitless mode. The SPME fiber was inserted directly into the front inlet of the gas chromatograph and desorbed at 270°C for 2 min. The oven temperature was programmed as follows: 40°C for 2 min, 40°C to 180°C at a rate of 4°C/min, 180°C to 250°C at a rate of 5°C/min, and holding at 250°C for 6 min. The temperature of the transfer line and ion trap were 150°C and 250°C, respectively. The identification of VOCs was based on a comparison of the mass spectrum of the substance with standards in the NIST08.L GC-MS system data bank (National Institute of Standards and Technology). The experiment was conducted three times.

### Analysis of the expression of STR-2 in AWC neurons.

The *kyIs140* transgenic mutant strain expressing STR-2::GFP was used to examine the expression level of STR-2 in AWC neurons (18). Young adult C. elegans worms were incubated in distilled water or FAc (0.2 mg/mL) at 20°C for 45 min, washed 3 times in distilled water, and then imaged under an Olympus BX63 microscope. The excitation and emission wavelengths were 489 nm and 508 nm, respectively. Worms treated with 2-heptanone (0.2 mg/mL) served as the positive controls. The experiment was repeated 3 times, and each treatment included at least 5 nematodes.

### Protein expression and purification.

The sequences of the extracellular domains of the STR-2 protein and SRA-13 isoform A and B were predicted using the TMHMM server (http://www.cbs.dtu.dk/services/TMHMM/) (47). All of the genes of the extracellular domain of the GPCR STR-2 were sequentially linked and synthesized to create the *str-2o* gene. Similarly, all of the genes of the extracellular domains of the GPCR SRA-13 isoform A or B were sequentially linked and synthesized to create the *sra-13ao* or *sra-13bo* gene. These genes (*str-2o*, *sra-13ao*, and *sra-13bo*) were synthesized by TsingKe (Beijing, China) and cloned into the vector pET28a to create the recombinant vectors pET28a-str2o, pET28a-sra13ao, and pET28a-sra13bo, respectively. The gene sequences of *str-2o*, *sra-13ao*, and *sra-13bo* are listed in Table S1 in the supplemental material. These recombinant plasmids were transformed into E. coli BL21(DE3) cells, and positive transformants were selected on LB plates containing 50 μg/mL kanamycin. The overexpression of these proteins was induced with isopropyl-β-d-thiogalactopyranoside (IPTG) in E. coli, and the proteins were purified using Ni-NTA.

### Measurement of binding by MST *in vitro*.

The purified potential target proteins were labeled with 2nd-generation RED-NHS according to the manufacturer’s instructions (Nano Temper, Germany). The labeled protein samples were diluted with phosphate-buffered saline (PBS) buffer (137 mM NaCl, 2.7 mM KCl, 10 mM Na_2_HPO_4_, and 2 mM KH_2_PO_4_ [pH 7.4]) to ensure that the fluorescence values were between 200 and 1500 counts. FAc was dissolved in PBS buffer, and 16 different concentrations were prepared by serial dilution. After mixing the dilutions with the labeled protein at a ratio of 1:1 for 10 min at 25°C, each sample was poured into a Monolith NT.115 standard capillary. The measurements were performed at 100% LED power (except for SRA-13AO at 80% LED power) and 40% MST power at 25°C. All MST binding experiments were performed at least three times, and the protein SUMO tag was used as a control. The data were analyzed by MO.Affinity analysis, and the *K_d_* values were calculated according to the binding curve (48, 49).

### Statistical analysis.

All data were analyzed by using SPSS (Statistical Package for the Social Sciences) version 22.0 software (SPSS, Chicago, IL, USA) and are shown as the means ± standard errors of the means (SEM). The significance of the differences between two groups was assessed by two-tailed unpaired Student’s *t* test, and a *P* value of <0.05 was considered significant.
