# Supplementary material for: Multiple Receptors Contribute to the Attractive Response of Caenorhabditis elegans to Pathogenic Bacteria
Source: Microbiol Spectr. 2022 Dec 13;11(1):e02319-22. doi: 10.1128/spectrum.02319-22 (PMC9927473; doi:10.1128/spectrum.02319-22)
Supplement: Supplemental file 1 — Supplemental material. Download spectrum.02319-22-s0001.pdf, PDF file, 0.9 MB [file spectrum.02319-22-s0001.pdf]

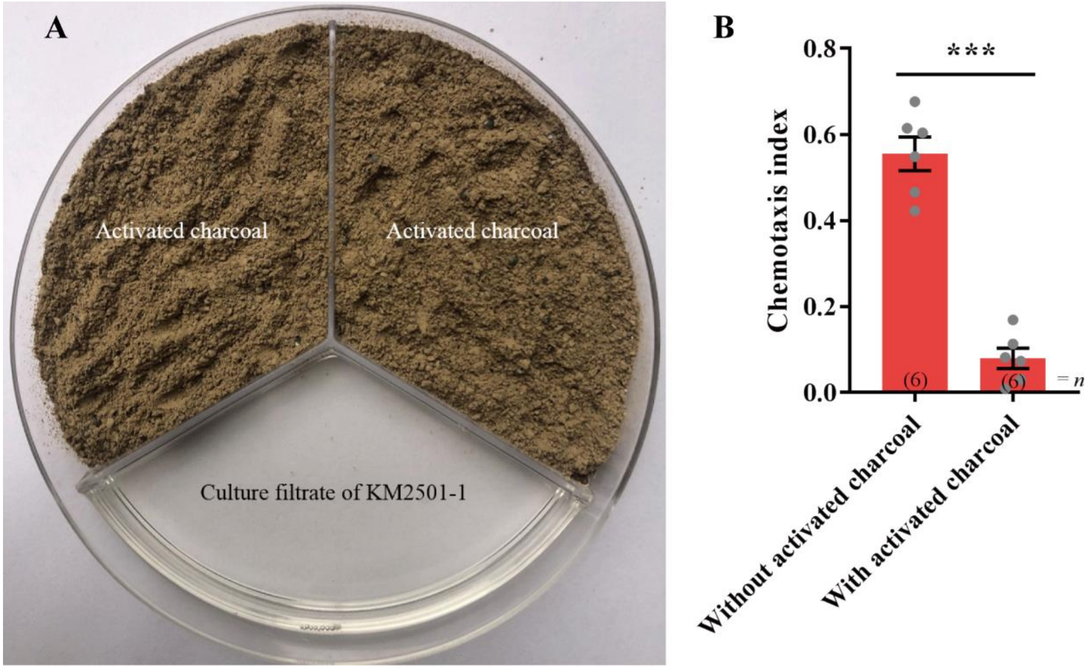

Figure S1. *Paenibacillus polymyxa* KM2501-1 elicits attractive response in *C. elegans* via volatile metabolites. (A) A three-compartment Petri plate (85 mm diameter) was used to remove the volatile substances in the culture filtrate of strain KM2501-1 using activated charcoal. In total of 4 mL of the original culture filtrate of strain KM2501-1 was added into one compartment, and 10 g of activated charcoal was introduced into other two compartments. Then the three-compartment Petri plate was sealed and maintained in 28°C for 48 h, the culture filtrate of strain KM2501-1 without treating with activated charcoal in three-compartment Petri plate was used as the control. (B) Chemotactic response of wild-type *C. elegans* towards original culture filtrate of strain KM2501-1 after treating with activated charcoal or not. Error bars indicate mean ± SEM. ***$P < 0.001$; a two-tailed unpaired Student's t test was used for statistical comparison between different treatments.

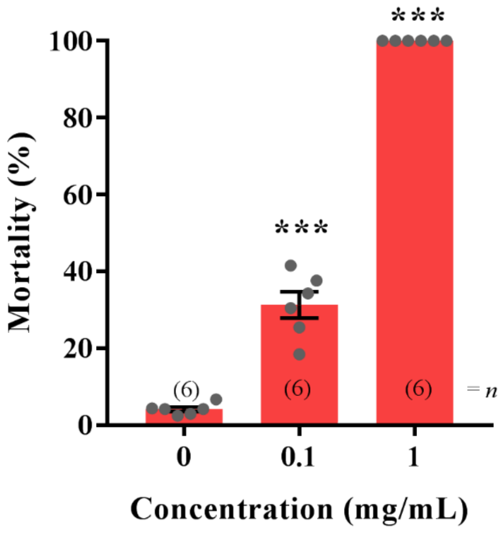

Figure S2. Nematicidal activity of FAc against wild-type *C. elegans* for 24 h. Error bars indicate mean ± SEM. ****P* < 0.001; a two-tailed unpaired Student's *t* test was used for statistical comparison between the values of the treatments and the control (0 mg/mL).

A
```
# SRA-13A Length: 335
# SRA-13A Number of predicted TMHs:  7
# SRA-13A Exp number of AAs in TMHs: 150.77796
# SRA-13A Exp number, first 60 AAs:  24.00063
# SRA-13A Total prob of N-in:        0.03982
# SRA-13A POSSIBLE N-term signal sequence
SRA-13A TMHMM1.0      outside     1    24
SRA-13A TMHMM1.0      TMhelix    25    47
SRA-13A TMHMM1.0      inside     48    58
SRA-13A TMHMM1.0      TMhelix    59    81
SRA-13A TMHMM1.0      outside    82   107
SRA-13A TMHMM1.0      TMhelix   108   126
SRA-13A TMHMM1.0      inside    127   146
SRA-13A TMHMM1.0      TMhelix   147   169
SRA-13A TMHMM1.0      outside   170   192
SRA-13A TMHMM1.0      TMhelix   193   215
SRA-13A TMHMM1.0      inside    216   243
SRA-13A TMHMM1.0      TMhelix   244   266
SRA-13A TMHMM1.0      outside   267   280
SRA-13A TMHMM1.0      TMhelix   281   299
SRA-13A TMHMM1.0      inside    300   335
```

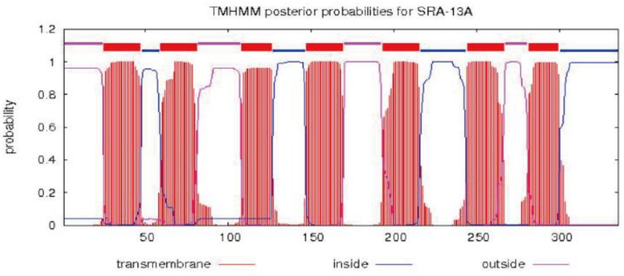

B
```
# SRA-13B Length: 174
# SRA-13B Number of predicted TMHs:  2
# SRA-13B Exp number of AAs in TMHs: 64.50199
# SRA-13B Exp number, first 60 AAs:  20.70724
# SRA-13B Total prob of N-in:        0.01517
# SRA-13B POSSIBLE N-term signal sequence
SRA-13B TMHMM1.0      outside     1    39
SRA-13B TMHMM1.0      TMhelix    40    62
SRA-13B TMHMM1.0      inside     63    73
SRA-13B TMHMM1.0      TMhelix    74    96
SRA-13B TMHMM1.0      outside    97   174
```

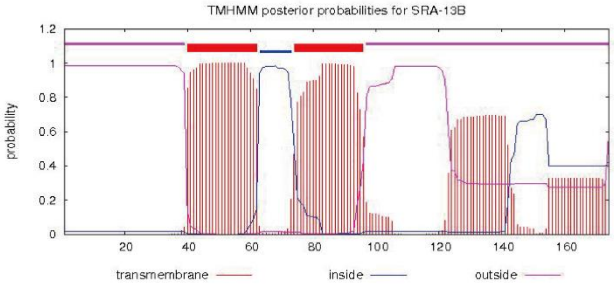

C
```
# STR-2 Length: 358
# STR-2 Number of predicted TMHs:  7
# STR-2 Exp number of AAs in TMHs: 154.81733
# STR-2 Exp number, first 60 AAs:  30.64303
# STR-2 Total prob of N-in:        0.01123
# STR-2 POSSIBLE N-term signal sequence
STR-2   TMHMM1.0      outside     1    14
STR-2   TMHMM1.0      TMhelix    15    33
STR-2   TMHMM1.0      inside     34    45
STR-2   TMHMM1.0      TMhelix    46    68
STR-2   TMHMM1.0      outside    69    92
STR-2   TMHMM1.0      TMhelix    93   115
STR-2   TMHMM1.0      inside    116   134
STR-2   TMHMM1.0      TMhelix   135   157
STR-2   TMHMM1.0      outside   158   202
STR-2   TMHMM1.0      TMhelix   203   225
STR-2   TMHMM1.0      inside    226   255
STR-2   TMHMM1.0      TMhelix   256   278
STR-2   TMHMM1.0      outside   279   292
STR-2   TMHMM1.0      TMhelix   293   311
STR-2   TMHMM1.0      inside    312   358
```

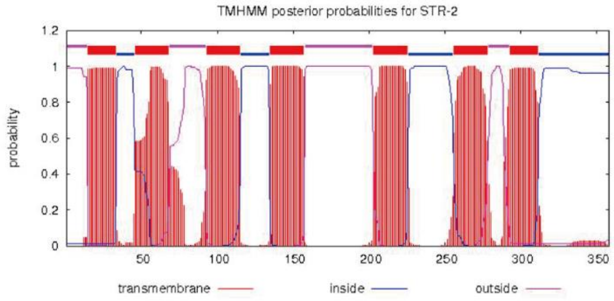

Figure S3. Prediction of transmembrane protein topology by the TMHMM server.

(A) Protein SRA-13 isoform A (SRA-13A). (B) Protein SRA-13 isoform B (SRA-13B).

(C) Protein STR-2.

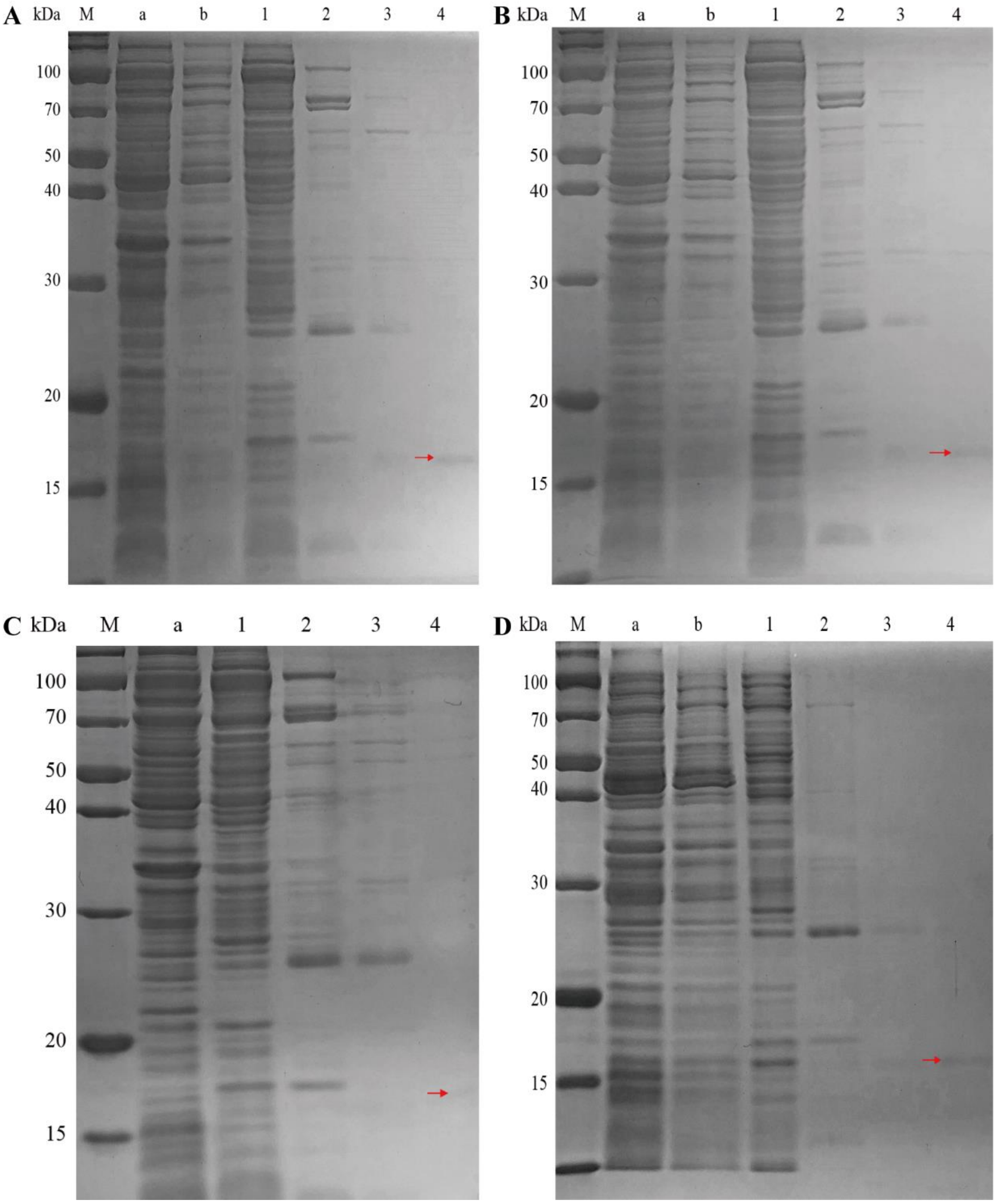

Figure S4. Purified the extracellular domains of potential target protein of FAc in AWA and AWC neurons. (A) SRA-13AO, (B) SRA-13BO, (C) STR-2O, and (D) SUMO. M, marker; Lane a: *E. coli* BL21 induced with 0.5 mM IPTG at 22°C overnight; lane b: purified protein eluted from PBS buffer; lanes 1-4, purified protein eluted from 20, 50, 100 and 500 mM imidazole dissolved in PBS buffer, respectively. Red arrowhead indicate the band of purified protein.

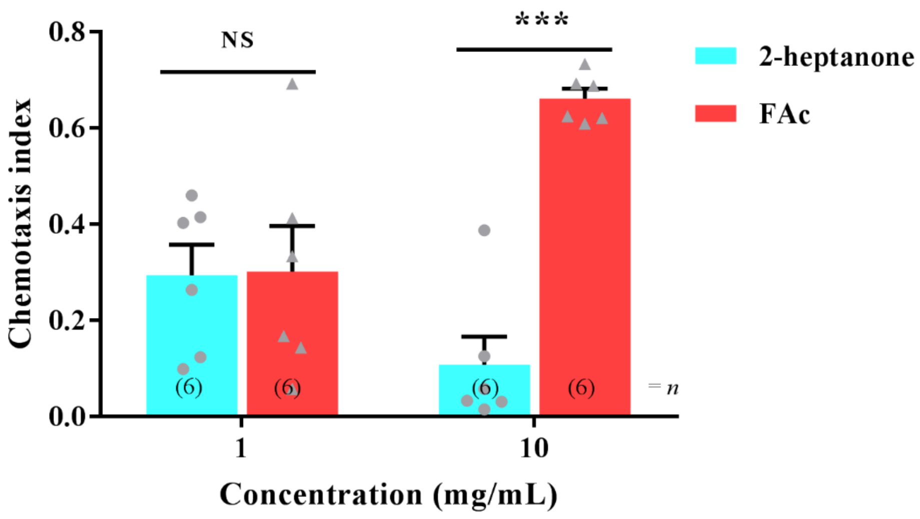

Figure S5. Chemotaxis effect of FAc and 2-heptanone on the wild-type *C. elegans*. Error bars indicate mean ± SEM. ****P* < 0.001; NS, not significant; a two-tailed unpaired Student's *t* test was used for statistical comparison between the values of the FAc treatment and the 2-heptanone treatment.

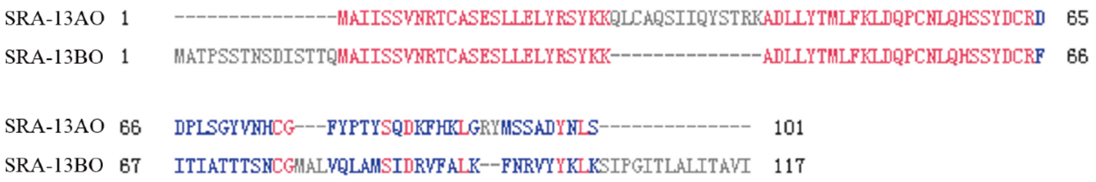

Figure S6. Sequence alignment analysis of SRA-13AO and SRA-13BO was performed by protein-protein BLAST in NCBI. The red color indicates sequence matched.

Table S1: The gene sequence of genes *sra-13ao*, *sra-13bo*, and *str-2o*

Genes	Sequence
sra-13ao	gtcgacccATGGCTATTATCAGTTCTGTCAACCGGACATGTGCTTCCGAATCTCTTCTTGAGCTGTACCGTTCATATAAAAAGCAGTTATGTGCACAGTCTATTATACAATACTCGACAAGAAAGGCTGACCTACTCTACACGATGCTTTTCAAATTAGATCAACCTTGCAATCTTCAACATTCTTCCTATGATTGCCGGGATGATCCACTGTCAGGATACGTAAATCATTGTGGTTTCTATCCAACATATTCTCAAGATAAATTTCATAAGTTGGGAAGATATATGTCATCTGCTGATTACAACCTTTCCgcggccgc
sra-13bo	gtcgacccATGGCAACTCCTTCCTCAACTAACTCAGATATTTCCACTACTCAAATGGCTATTATCAGTTCTGTCAACCGGACATGTGCTTCCGAATCTCTTCTTGAGCTGTACCGTTCATATAAAAAGGCTGACCTACTCTACACGATGCTTTTCAAATTAGATCAACCTTGCAATCTTCAACATTCTTCCTATGATTGCCGGTTTATCACAATTGCAACAACAACATCCAACTGTGGGATGGCTCTTGTTCAACTTGCAATGTCTATTGACCGAGTTTTTGCACTGAAGTTCAACAGAGTTTACTACAAACTCAAATCGATTCCGGGGATAACTTTAGCTTTAATTACTGCGGTCATAgcggccgc
str-2o	gtcgacccATGCCGACTGTGCAATGGATTCAGCTGGAAGAGACTTCCAAACACACATATGGGTCTGCTTTTATTGTATTTCAAGACATGAACACATTTTACTTCAGTCATCATGTTGCACAAGAAACGCCGGATAGAACCAACTATTTGAGGGAAACCTTGCTCAGCAACTACCGATTGAAAATTGAAGAATGTGCATACATTAGTGCTCGTTTCTGGGTCTCAGATAAGAATAACTACTTGTTTCCGGACTTCGATCCGATGCTTAACACAGAACTTAATTTGAAATATCCTTTCATTgcggccgc
